# Evaluation of the clinical effectiveness of bundled care interventions on pressure ulcer incidence in neurosurgical patients

**DOI:** 10.3389/fpubh.2025.1576633

**Published:** 2025-06-10

**Authors:** Ai-Hua Lv, Xi Zhang, Xiu-Ling Sun, Xiu-Lan Liu, Jun-Qing Qi, Hui-Na Li

**Affiliations:** ^1^Department of Neurosurgery, The First Affiliated Hospital of Xinxiang Medical College, Xinxiang, China; ^2^Department of Neurology, The First Affiliated Hospital of Xinxiang Medical College, Xinxiang, China; ^3^Department of Neurosurgery Intensive Care Unit, The First Affiliated Hospital of Xinxiang Medical College, Xinxiang, China; ^4^Department of Nursing, The First Affiliated Hospital of Xinxiang Medical College, Xinxiang, China

**Keywords:** pressure ulcers, bundled care interventions, neurosurgery, post-operative care, Waterlow scale

## Abstract

**Background:**

Pressure ulcers are a common and serious complication in neurosurgical patients, primarily due to prolonged immobility. Effective prevention is essential to improving patient outcomes. This study aimed to evaluate the clinical effectiveness of bundled care interventions in reducing the incidence of pressure ulcers and improving quality of life and nursing satisfaction in neurosurgical patients.

**Methods:**

This retrospective study included 316 neurosurgical patients, divided into two groups: the study group (*n* = 158) received bundled care interventions, while the control group (*n* = 158) received conventional care, which involved standard post-operative interventions such as regular repositioning, pressure-relieving devices, health education, and psychological support. Bundled care included pre-operative risk assessment using the Waterlow Scale, targeted preventive measures, health education, nutritional support, and post-operative monitoring. Data were collected on pressure ulcer incidence, quality of life (assessed by the Short Form Health Survey), nursing satisfaction, and length of hospital stay. Statistical analysis was performed using SPSS Version 27.0, applying independent sample *t*-tests and chi-square tests where appropriate.

**Results:**

The study group demonstrated a significantly lower incidence of pressure ulcers (11.39%) compared to the control group (19.62%, *χ*^2^ = 4.08, *p* < 0.05). The study group also exhibited significantly improved quality of life outcomes across all domains, including physical, physiological, emotional, and social functioning (*p* < 0.001 for all). In physical functioning, the study group scored 83.33 ± 2.12, while the control group scored 77.20 ± 1.89. Emotional functioning also improved significantly, with the study group scoring 85.32 ± 2.05 compared to 76.41 ± 2.12 in the control group. Nursing satisfaction was higher in the study group, with an overall satisfaction rate of 96.20%, compared to 88.61% in the control group (*χ*^2^ = 6.49, *p* < 0.05). The study group reported a significantly lower proportion of dissatisfied patients and a higher proportion of those who were highly satisfied.

**Conclusion:**

Bundled care interventions significantly reduce pressure ulcer incidence and improve quality of life and nursing satisfaction in neurosurgical patients. This multidisciplinary approach effectively addresses both physical and psychological aspects of post-operative care, contributing to better patient outcomes and overall satisfaction.

## 1 Introduction

Pressure ulcers, also known as decubitus ulcers or bedsores, represent a significant clinical challenge, particularly in vulnerable patient populations such as those undergoing neurosurgical procedures. These injuries, caused by prolonged pressure on the skin and underlying tissues, often exacerbated by contact with a hard surface, can lead to serious complications, including infection, prolonged hospital stays, and increased healthcare costs (1). Neurosurgical patients are at especially high risk due to factors such as immobility, altered levels of consciousness, and the need for extended bed rest. The prevention and management of pressure ulcers in this population require targeted, evidence-based interventions to mitigate these risks and improve patient outcomes (2–4). In recent years, bundled care interventions have emerged as a promising strategy to reduce the incidence of pressure ulcers in high-risk patient groups. A “bundle” in healthcare refers to a set of evidence-based practices that, when implemented together, yield better outcomes than when applied individually. Bundled care interventions for pressure ulcer prevention typically include a combination of regular skin assessments, repositioning protocols, nutritional support, and the use of pressure-relieving devices such as specialized mattresses or cushions (5, 6). The premise behind bundled care is that the cumulative effect of these interventions is greater than the sum of its parts, creating a more comprehensive approach to patient care that addresses multiple facets of pressure ulcer prevention simultaneously.

The neurosurgical population presents unique challenges in terms of pressure ulcer prevention. Due to the nature of their conditions, these patients often experience prolonged immobility, which significantly increases their risk of developing pressure ulcers. Furthermore, their neurological status may impair their ability to communicate discomfort, making it more difficult for healthcare providers to identify early signs of pressure injury (7, 8). Additionally, the use of medical devices such as cervical collars, intracranial pressure monitors, or external ventricular drains can further exacerbate pressure on certain areas of the body, increasing the likelihood of ulcer formation. Therefore, tailored interventions are crucial to prevent these complications. Several studies have demonstrated the efficacy of bundled care interventions in reducing pressure ulcer incidence across various patient populations, but there remains a need for focused research on their application in neurosurgical patients. Given the complexity of care required for these patients, it is critical to understand how bundled care strategies can be optimized for this specific group (9–11). Bundled interventions that are carefully designed and consistently implemented have the potential to significantly reduce the incidence of pressure ulcers, thereby improving clinical outcomes and reducing the overall burden on healthcare systems.

This study aims to evaluate the clinical effectiveness of bundled care interventions on the incidence of pressure ulcers in neurosurgical patients. By focusing on this high-risk population, the study seeks to identify key components of bundled care that contribute most significantly to the prevention of pressure ulcers and to provide evidence for the broader application of these interventions in clinical practice. The findings from this research may guide the development of more effective care protocols, ultimately leading to improved patient safety and reduced complications in neurosurgical care settings.

## 2 Methods

### 2.1 Study design

A comprehensive retrospective evaluation was undertaken at our institution to assess the clinical efficacy of bundled care interventions in reducing the incidence of pressure ulcers among neurosurgical patients. This study spanned a period from December 2018 to December 2023. A cohort of 158 patients who underwent bundled care interventions was selected for the observational group for detailed analysis. To enable a meaningful comparison, a control group was also constituted, comprising 158 patients from the same timeframe who received standard care interventions, thus ensuring the comparability of the two groups. The study was approved by the Ethics Committee of the First Affiliated Hospital of Xinxiang Medical College. The design and execution conformed to the ethical standards outlined in the Declaration of Helsinki for research involving human subjects. Informed consent was obtained from all participants or their legal guardians for participation, and consent for the publication of data was provided by the patients and/or their families via telephone. Data confidentiality was strictly maintained, with all personal identifiers removed to ensure privacy.

### 2.2 Inclusion and exclusion criteria

Inclusion criteria: (1) Age: Patients aged 18 years or older, regardless of gender; (2) Surgical Status: Patients who have undergone any form of neurosurgical procedure, including but not limited to craniotomy, spinal surgery, or any other invasive neurosurgical intervention; (3) Hospitalization Duration: Patients with an expected hospital stay of at least 7 days postoperatively; (4) Consent: Patients or their legal representatives who provide informed consent to participate in the study.

Exclusion criteria: (1) Pre-existing Pressure Ulcers: Patients presenting with stage II or higher pressure ulcers at the time of neurosurgical admission. (2) Non-Neurosurgical Conditions: Patients admitted for non-neurosurgical reasons, even if they are under the care of a neurosurgical team. (3) Short Hospitalization: Patients with an anticipated hospital stay of less than 72 h. (4) Medical Instability: Patients with severe medical instability or terminal illness where pressure ulcer prevention is not a primary clinical focus. (5) Incomplete Data: Patients with incomplete medical records or missing data critical for the study’s outcome analysis.

### 2.3 Nursing protocols for the control group and observation group

In the control group, patients received conventional post-operative care interventions tailored to their individual needs, encompassing medication guidance, health education, dietary interventions, psychological counseling (provided upon request by patients or family members due to emotional concerns), and interventions to prevent complications or adverse events associated with pressure ulcers.

In the observation group received bundled care interventions based on the Waterlow Pressure Ulcer Risk Assessment Scale, detailed as follows:

1. Risk assessment: Clinical nursing staff utilized the Waterlow Scale a day before surgery to comprehensively evaluate patients on factors such as gender, age, body mass, appetite, nutritional status, mental state, and surgical duration, with scores ≥20 indicating very high risk, 15–19 high risk, and 10–14 moderate risk of pressure injury.
2. Surgical measures based on risk assessment: For patients at moderate risk, enhanced care was provided to critical areas such as the knees and shoulders, with patients positioned laterally. For those at high risk, care intensification at crucial sites was combined with keeping the patient’s limbs in a relaxed position and periodic adjustments to the operating table’s inclination. For patients at very high risk, immediate reporting to the senior nursing department was required for a targeted consultative approach, with vigilant post-operative monitoring of pressure areas and preemptive interventions.
3. Bundled care health education: Prior to surgery, clinical nursing staff educated patients and their families about the causes and prevention of pressure ulcer, aiming to enhance awareness and self-protection. Families were instructed on techniques for repositioning the patient and simple massage methods for critical areas.
4. Enhanced nutritional support in bundled care: Recognizing the extensive physical trauma experienced by patients, prompt supplementation with proteins, vitamins, and other nutrients was advised, tailored to the patient’s financial circumstances.
5. Post-operative monitoring of pressure ulcer: Patients were closely monitored post-operatively based on their pre-operative Waterlow scale categorization, with targeted pressure injury prevention nursing care implemented according to the risk level.
6. Care of pressure areas: Nursing staff were required to assist patients with regular body cleaning, repositioning, and frequent changes of bed linens and clothing to maintain a clean and comfortable environment. Massaging pressure areas was recommended to prevent venous thrombosis and alleviate discomfort.
7. Psychological interventions: Given the significant emotional fluctuations experienced by neurosurgical patients confined to prolonged bed rest, healthcare professionals employed positive and reassuring communication to soothe and motivate patients, helping them relax and divert their focus.

### 2.4 Data collection

The data collection process encompassed four primary domains: the incidence of pressure ulcer, quality of life assessments, nursing satisfaction levels, and length of hospital stay. Pressure injury incidence was meticulously documented for each patient, calculating the rate of occurrence and facilitating comparative analyses. Quality of life was evaluated using the Short Form Health Survey (SF-36), which includes domains such as physical, psychological, cognitive, and social functioning, with each domain scored from 0 to 100. Higher scores are indicative of a better quality of life. Nursing satisfaction was gaged through a standardized Nursing Satisfaction Survey, scored out of 100, where scores above 90 denoted high satisfaction, 80–90 indicated satisfaction, 60–79 reflected basic satisfaction, and scores below 60 represented dissatisfaction. Overall satisfaction percentage was calculated as the sum of satisfied and highly satisfied cases divided by the total number of cases, multiplied by 100%. Additionally, the length of hospital stay for each patient was recorded, allowing for further analysis of recovery outcomes and healthcare resource utilization. In addition, we collected detailed baseline demographic and clinical variables. These included: age, gender, type of neurosurgical procedure, and Body mass index (BMI). These were extracted from the electronic medical records to ensure comparability between the observation and control groups at baseline. All data were reviewed to confirm completeness and accuracy prior to analysis.

### 2.5 Statistical analysis

Statistical evaluation was rigorously conducted employing SPSS Version 27.0. Initially, data were segregated into quantitative and categorical variables, followed by the implementation of normality assessments to determine their distribution characteristics. For quantitative variables adhering to normal distribution, comparisons between groups were facilitated using independent sample *t*-tests, with findings depicted as mean ± standard deviation. Conversely, for quantitative variables deviating from normal distribution, data were represented using medians and interquartile ranges (M[P25, P75]), and inter-group comparisons were conducted utilizing the Mann–Whitney *U* test. Categorical variables were articulated as counts and percentages, with Chi-square (*χ*^2^) tests applied to examine the independence or relationships between these variables. The study adopted a two-tailed hypothesis testing approach, setting a *p*-value of less than 0.05 as the criterion for establishing statistical significance. To account for potential confounding factors, we performed an additional binary logistic regression analysis to evaluate the association between care model (bundled vs. conventional) and pressure ulcer incidence. Adjusted odds ratios (aORs) with 95% confidence intervals (CIs) were reported. A two-tailed *p* value < 0.05 was considered statistically significant.

## 3 Results

### 3.1 Clinical baseline characteristics

A total of 316 patients were included in this study, with 158 patients in the bundled care group (study group) and 158 patients in the conventional care group (control group). The mean age of patients was comparable between the groups: 43.86 ± 7.86 years in the bundled care group (range, 23–78 years) and 44.01 ± 8.16 years in the control group (range, 21–81 years). Gender distribution was balanced, with 50.6% males and 49.4% females in the bundled care group, and 51.9% males and 48.1% females in the control group. Regarding the type of neurosurgical procedures, craniotomy was the most common intervention (57.6% in the bundled care group and 58.9% in the control group), followed by spinal surgery (34.2 and 32.9%, respectively), and other neurosurgical procedures (8.2% in each group). Body mass index (BMI) was similar between the two groups, with mean values of 23.73 ± 2.94 kg/m^2^ and 23.89 ± 3.01 kg/m^2^ in the bundled and conventional care groups, respectively. Comparative analysis of these baseline demographic and clinical parameters revealed no statistically significant differences between groups (all *p* > 0.05), indicating that the two groups were well-matched at baseline and suitable for subsequent comparative analyses (Table 1).

**Table 1:** Baseline characteristics of patients.

Variable	Bundled care group (*n* = 158)	Conventional care group (*n* = 158)	*p*-value
Age (years), mean ± SD	43.86 ± 7.86	44.01 ± 8.16	>0.05
Age range (years)	23–78	21–81	—
Gender, *n* (%)			>0.05
Male	80 (50.6%)	82 (51.9%)	
Female	78 (49.4%)	76 (48.1%)	
Type of neurosurgical procedure, n (%)			>0.05
Craniotomy	91 (57.6%)	93 (58.9%)	
Spinal surgery	54 (34.2%)	52 (32.9%)	
Others	13 (8.2%)	13 (8.2%)	
BMI (kg/m^2^), mean ± SD	23.73 ± 2.94	23.89 ± 3.01	>0.05

### 3.2 Comparison of pressure ulcer incidence between the two groups

**Figure 1:**
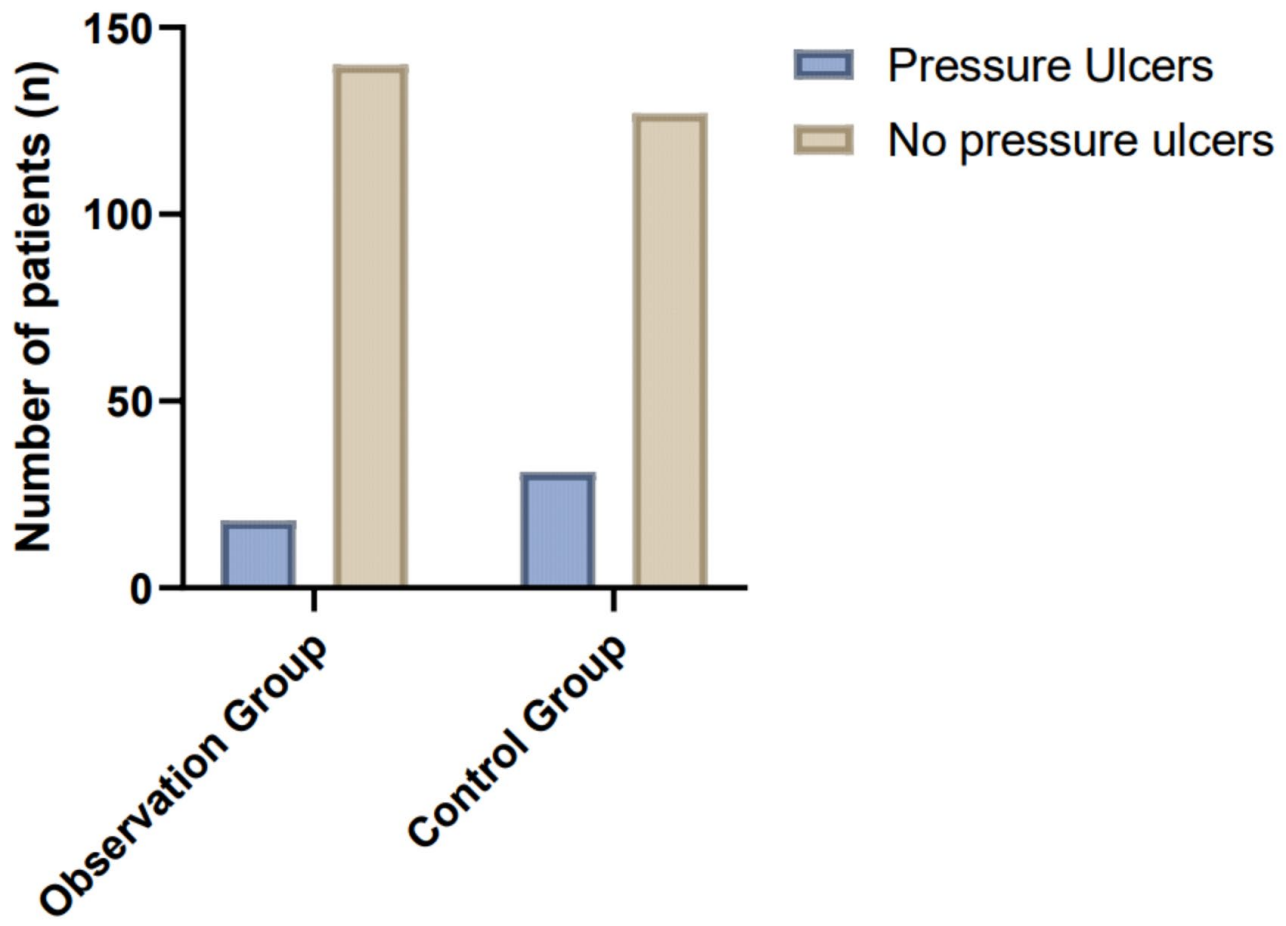
Comparison of pressure ulcer incidence in the study and control groups.

In this study, the incidence of pressure ulcers was significantly different between the two groups. In the study group, which received bundled care interventions, 18 cases of pressure ulcers were documented, resulting in an incidence rate of 11.39%. In contrast, the control group, which received conventional post-operative care, experienced 31 cases of pressure ulcers, with an incidence rate of 19.62%. Statistical analysis using the chi-square test (*χ*^2^ = 4.08) revealed that the difference in pressure ulcer incidence between the two groups was statistically significant (*p* < 0.05) (Table 2; Figure 1). These findings indicate that the implementation of bundled care interventions significantly reduced the risk of developing pressure ulcers compared to conventional care.

**Table 2:** Comparison of pressure ulcer incidence between the study and control groups.

Group	Cases observed (*n*)	No pressure ulcers (*n*)	Pressure ulcer cases (n)
Observation group	158	140	18
Control group	158	127	31
*χ*^2^ value	-	-	4.08
*p* value	-	-	<0.05

To further validate the findings, a multivariate logistic regression analysis was conducted with pressure ulcer occurrence as the dependent variable and group assignment, age, gender, BMI, and procedure type as independent variables. The analysis confirmed that bundled care remained an independent protective factor against pressure ulcer development (adjusted OR = 0.54, 95% CI: 0.29–0.99, *p* = 0.047), indicating that the observed group difference was not attributable to baseline covariates.

### 3.3 Comparison of quality of life between the study and control groups

**Figure 2:**
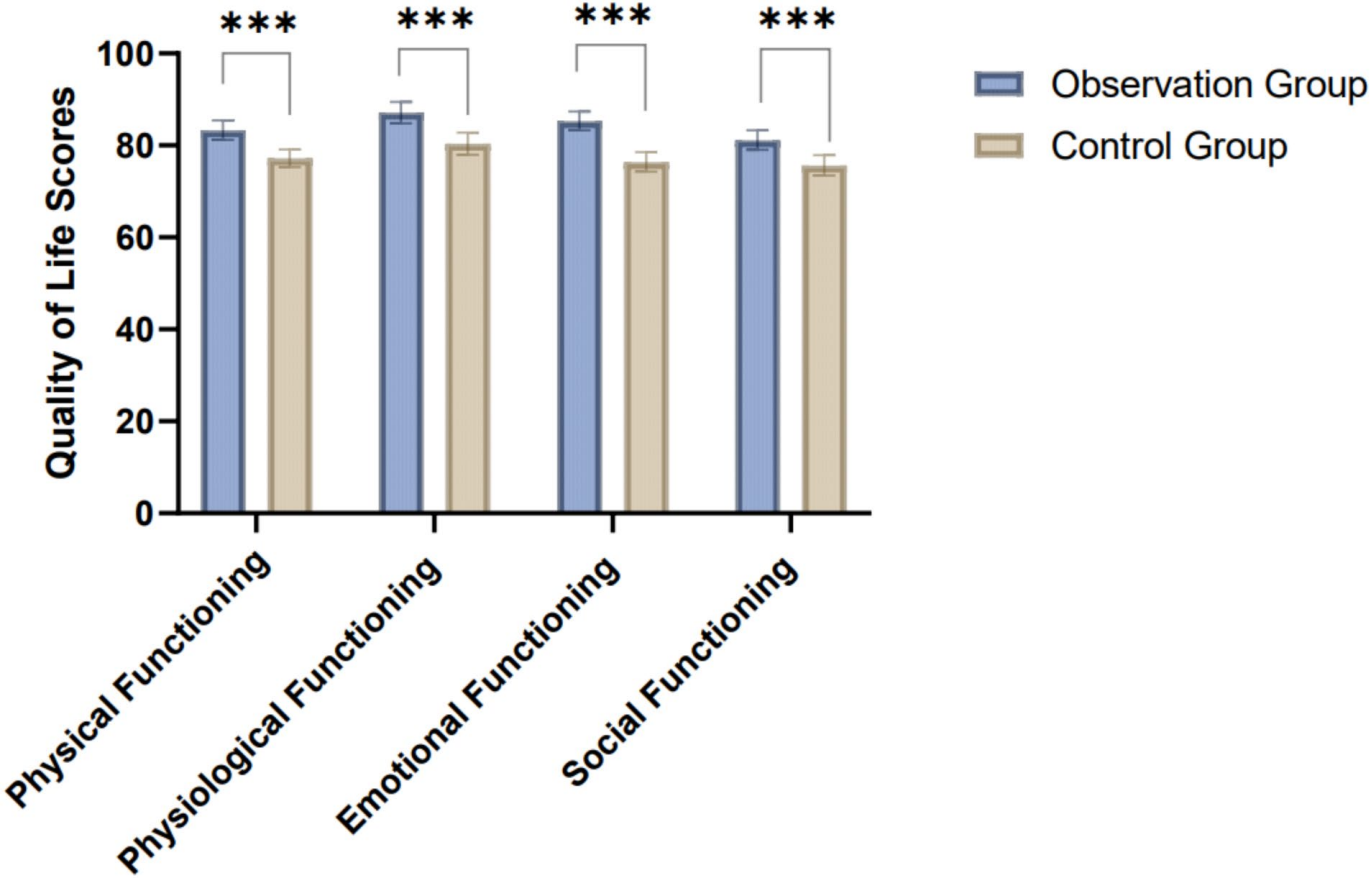
Comparison of quality of life scores in the study and control groups.

The results show that the observation group, which received bundled care interventions, exhibited significantly better quality of life outcomes across all domains compared to the control group. In physical functioning, the observation group had a higher score (83.33 ± 2.12) than the control group (77.20 ± 1.89), with a significant difference (*t* = 27.13, *p* < 0.001). Similarly, in physiological functioning, the observation group scored 87.13 ± 2.35, compared to 80.40 ± 2.41 in the control group (*t* = 25.13, *p* < 0.001). Emotional functioning also improved, with scores of 85.32 ± 2.05 for the observation group and 76.41 ± 2.12 for the control group (*t* = 37.98, *p* < 0.001). Social functioning showed similar trends, with the observation group scoring 81.16 ± 2.14, significantly higher than the control group (75.66 ± 2.24; *t* = 22.32, *p* < 0.001) (Table 3; Figure 2). These results demonstrate the effectiveness of bundled care in improving post-operative recovery and overall quality of life in neurosurgical patients.

**Table 3:** Comparison of quality of life scores between the study and control groups (Mean ± SD).

Group	Physical functioning	Physiological functioning	Emotional functioning	Social functioning
Observation group	83.33 ± 2.12	87.13 ± 2.35	85.32 ± 2.05	81.16 ± 2.14
Control group	77.20 ± 1.89	80.40 ± 2.41	76.41 ± 2.12	75.66 ± 2.24
*t* value	27.13	25.13	37.98	22.32
*p* value	<0.001	<0.001	<0.001	<0.001

### 3.4 Comparison of nursing satisfaction between the study and control groups

The results indicate a significant difference in nursing satisfaction between the study group, which received bundled care interventions, and the control group. The overall satisfaction rate in the study group was 96.20%, significantly higher than the control group, which had a satisfaction rate of 88.61%. The study group had fewer dissatisfied patients (6) compared to the control group (18), with more patients reporting “satisfied” and “highly satisfied” ratings in the study group (58 and 66, respectively) than in the control group (39 and 45, respectively). Statistical analysis using the chi-square test (*χ*^2^ = 6.49, *p* < 0.05) confirmed that the differences in nursing satisfaction between the two groups were statistically significant. These findings suggest that the implementation of bundled care interventions not only improves clinical outcomes but also significantly enhances patient and family satisfaction with the nursing care provided during the post-operative recovery period (Table 4).

**Table 4:** Comparison of nursing satisfaction between the study and control groups.

Group	Dissatisfied (*n*)	Basic satisfaction (*n*)	Satisfied (*n*)	Highly satisfied (*n*)	Satisfaction rate (%)
Observation group	6	28	58	66	96.20
Control group	18	56	39	45	88.61
*χ*^2^ value	-	-	-	-	6.49
*p* value	-	-	-	-	<0.05

### 3.5 *Post-hoc* weighted power analysis

A *post-hoc* power analysis was conducted for the six predefined outcome variables: pressure ulcer incidence, physical functioning, physiological functioning, emotional functioning, social functioning, and nursing satisfaction. Using a weighted average approach across these outcomes, the overall statistical power was calculated to be 0.88. This exceeds the conventional threshold of 0.80, indicating that the study had sufficient power to detect the observed effects at a significance level of *α* = 0.05 (two-tailed), assuming equal importance across outcomes.

## 4 Discussion

Numerous studies have explored the use of structured or bundled nursing interventions to prevent pressure injuries across various clinical settings, providing important context for the interpretation of our findings (12–14). Jiang et al. (15) compared CORN-based intraoperative nursing with routine care in neurosurgical patients and demonstrated a significant reduction in intraoperative pressure injuries. While their study focused exclusively on the intraoperative phase, our findings extend these results by evaluating a bundled intervention encompassing pre-, intra-, and post-operative stages. Unlike their single-setting approach, our comprehensive care model addresses both physical and psychosocial dimensions, achieving superior outcomes in pressure ulcer prevention and patient satisfaction. Rivera et al. (16) implemented a pressure injury prevention bundle in a critical care setting, reporting a marked decrease in HAPI incidence. Consistent with their findings, our study also supports the effectiveness of bundled strategies. However, our bundle incorporated risk stratification via the Waterlow Scale, structured nutritional and psychological support, and focused specifically on neurosurgical patients, thereby enhancing applicability to a high-risk surgical population with extended immobility. Chaboyer et al. (17) conducted a meta-analysis on the effect of pressure injury prevention bundles in hospitalized patients, noting modest benefits but low-certainty evidence, primarily due to methodological limitations. In contrast, our study applied a clearly defined care bundle with contemporaneous controls, high completion rate, and multivariate adjustment, producing statistically robust and clinically meaningful reductions in pressure ulcer incidence and improvements in quality-of-life outcomes.

The most striking finding in this study was the significant reduction in pressure ulcer incidence in the study group, where bundled care interventions were applied. The incidence rate in the study group was 11.39%, compared to 19.62% in the control group. Pressure ulcers are a major complication in neurosurgical patients due to prolonged immobility, compromised neurological function, and other risk factors (18, 19). The success of the bundled care interventions in this context can be attributed to a few key factors. First, early risk assessment using the Waterlow Pressure Ulcer Risk Assessment Scale was integral to identifying high-risk patients before surgery. By categorizing patients based on their risk levels and implementing tailored preventive measures accordingly, care providers were able to preemptively mitigate the risk of pressure ulcer development. Early identification allowed for targeted interventions, such as the use of specialized mattresses, frequent repositioning, and enhanced skin care, which collectively reduced the risk of tissue breakdown. Second, multidisciplinary involvement in bundled care likely contributed to the observed reduction in pressure ulcers (20–22). The involvement of nurses, nutritionists, and physical therapists ensured that patients received comprehensive, multi-faceted care that addressed both physical and nutritional factors contributing to skin integrity. Enhanced nutritional support, particularly the timely administration of proteins and vitamins, may have played a crucial role in promoting skin health and preventing tissue damage in high-risk patients. Finally, consistent post-operative monitoring and the regular reassessment of patients’ risk status ensured that interventions were adapted as needed throughout the recovery process. This level of vigilance is often difficult to achieve with conventional care, where interventions may be less consistent or reactive rather than proactive (23, 24).

The study group, which received bundled care, exhibited significantly better quality of life outcomes across all domains, including physical, physiological, emotional, and social functioning. These results suggest that the bundled care interventions had a holistic impact on patients’ recovery, extending beyond the prevention of physical complications to encompass psychological and social well-being. The improvements in physical and physiological functioning (*p* < 0.001) are likely due to the comprehensive nature of the bundled care interventions, which included early mobilization, appropriate postural support, and effective management of potential complications such as pressure ulcers. These measures helped to maintain patients’ physical integrity, enabling them to regain mobility and reduce the length of time spent in bed, which is crucial for preventing further deconditioning and enhancing functional outcomes post-surgery. The significant improvements in emotional functioning (*p* < 0.001) can be attributed to the psychological support embedded in the bundled care protocol. Neurosurgical patients often experience significant emotional fluctuations due to the nature of their conditions and the stress associated with surgery and recovery. The inclusion of psychological counseling, coupled with health education for both patients and their families, likely alleviated emotional distress and promoted a more positive outlook during the recovery process (25, 26). This, in turn, could have facilitated better overall recovery, as emotional well-being is closely linked to physical health outcomes. The enhancement in social functioning (*p* < 0.001) observed in the study group can also be explained by the patient- and family-centered approach of the bundled care model. Involving families in patient education and recovery plans not only improved the quality of care but also helped patients maintain stronger social connections during their hospital stay (27, 28). This aspect of care is often overlooked in conventional protocols but is critical in promoting overall recovery and preventing feelings of isolation, especially in long-term hospitalized patients.

Nursing satisfaction was another important outcome that significantly differed between the two groups, with the study group showing a higher overall satisfaction rate (96.20%) compared to the control group (88.61%, *p* < 0.05). The improved nursing satisfaction in the bundled care group can be linked to several factors. Firstly, the structured nature of bundled care likely made the nursing staff’s workflow more efficient and focused. With clear guidelines and protocols for assessing and managing pressure ulcers and other post-operative risks, nurses may have experienced greater job satisfaction due to the improved clarity of their roles and responsibilities. This, combined with a reduction in pressure ulcer incidence, could have further enhanced satisfaction by reducing the burden of managing complications and ensuring that patients under their care experienced better outcomes (29, 30). Additionally, increased patient and family engagement may have contributed to higher satisfaction levels. When patients and families are well-informed and involved in the care process, it fosters a collaborative environment that can reduce the likelihood of complaints or misunderstandings about care quality. In turn, this positive feedback loop can improve nursing staff morale, as they are more likely to receive positive feedback from satisfied patients and families.

The success of the bundled care interventions in this study can be explained by their holistic and integrative approach. Bundled care addresses multiple facets of patient care, from early risk assessment and preventive measures to continuous post-operative monitoring and psychological support. This multi-dimensional approach ensures that patients receive consistent and personalized care, which is crucial in high-risk populations like neurosurgical patients. Moreover, the proactive nature of bundled care ensures that complications such as pressure ulcers are prevented rather than treated after they arise. This contrasts with conventional care, which may focus on reacting to problems rather than preventing them from occurring (31, 32). By addressing both physical and emotional aspects of patient care, bundled care creates a more comprehensive treatment plan that ultimately leads to better patient outcomes and higher satisfaction rates among both patients and healthcare providers.

This study has several limitations that should be acknowledged. First, its retrospective observational design introduces inherent risks of selection bias, as patients were allocated to groups based on the type of care received rather than through randomized assignment. Although efforts were made to ensure baseline comparability, the absence of randomization may limit causal inference. Second, the single-center setting may affect the external validity of the findings, as care protocols and patient characteristics may differ across institutions. Third, the relatively short follow-up period may not adequately capture long-term outcomes or delayed complications related to pressure ulcers. To enhance the robustness and generalizability of future research, prospective, multicenter randomized controlled trials with extended follow-up durations are warranted to confirm the sustained effectiveness of bundled care interventions across diverse clinical settings and patient populations.

## 5 Conclusion

The application of bundled care interventions in neurosurgical patients appears to be associated with a reduction in the incidence of pressure ulcers, as well as improvements in patients’ quality of life and overall satisfaction. This comprehensive, multidisciplinary approach may enhance post-operative care by addressing both physical and psychological needs, suggesting that bundled care could be a promising strategy for improving outcomes in high-risk neurosurgical populations. However, further prospective, multicenter studies are needed to confirm these findings and better understand the long-term effects.

## Data Availability

The raw data supporting the conclusions of this article will be made available by the authors, without undue reservation.

## References

[1] Qazi M Khattak AF Barki MT. Pressure ulcers in admitted patients at a tertiary care hospital. Cureus. (2022) 14:e24298. doi: 10.7759/cureus.24298, PMID: 35607569 PMC9123348

[2] Farahbakhsh F Rezaei Aliabadi H Baigi V Ghodsi Z Dashtkoohi M Pour-Rashidi A. Pressure ulcers and acute risk factors in individuals with traumatic spinal fractures with or without spinal cord injuries: a prospective analysis of the National Spinal Column/cord injury registry of Iran (NSCIR-IR) data. Chin J Traumatol. (2023) 26:193–8. doi: 10.1016/j.cjtee.2023.03.007, PMID: 37062622 PMC10388246

[3] Kim MS Ryu JM Choi BK. Development and effectiveness of a clinical decision support system for pressure ulcer prevention care using machine learning: a quasi-experimental study. Comput Inform Nurs. (2022) 9:899. doi: 10.1097/CIN.0000000000000899 35266901

[4] Behnammoghadam M Alimohammadi N Riazi A Eghbali-Babadi M Rezvani M. Incidence of cervical collar-related pressure injury in patients with head and neck trauma: a scoping review study. J Educ Health Promot. (2023) 12:252. doi: 10.4103/jehp.jehp_41_23, PMID: 37727424 PMC10506768

[5] Minteer DM Simon P Taylor DP Jia W Li Y Sun M. Pressure ulcer monitoring platform-a prospective, human subject clinical study to validate patient repositioning monitoring device to prevent pressure ulcers. Adv Wound Care. (2020) 9:28–33. doi: 10.1089/wound.2018.0934, PMID: 31871828 PMC6922059

[6] Luo M Long XH Wu JL Huang SZ Zeng Y. Incidence and risk factors of pressure injuries in surgical spinal patients: a retrospective study. J Wound Ostomy Continence Nurs. (2019) 46:397–400. doi: 10.1097/WON.0000000000000570, PMID: 31513127

[7] Pang D Liu Z Wang L. Comparison of nursing aids and registered nurses mixed nursing staffing model with different ratios on the nursing outcomes and cost in neurology and neurosurgery center. Ir J Med Sci. (2019) 188:1435–41. doi: 10.1007/s11845-019-01988-8, PMID: 30903451

[8] Donhauser M Grassner L Klein B Voth M Mach O Vogel M. Severe pressure ulcers requiring surgery impair the functional outcome after acute spinal cord injury. Spinal Cord. (2020) 58:70–7. doi: 10.1038/s41393-019-0325-x, PMID: 31312018

[9] Efteli E Güneş Ü. Assessing the validity and reliability of a new pressure ulcer risk assessment scale for patients in intensive care units. Wound Manag Prev. (2020) 66:24–33. doi: 10.25270/wmp.2020.2.2433, PMID: 32294059

[10] Yu G Sun C Hao S Wu H. Comparative analysis of pressure ulcer development in stroke patients within and outside healthcare facilities: a systematic review and meta-analysis. Int Wound J. (2024) 21:e14840. doi: 10.1111/iwj.14840, PMID: 38556516 PMC10982073

[11] Wu Y Jiang Z Huang S Shi B Wang C Zeng Y. Identification of risk factors for intraoperative acquired pressure injury in patients undergoing neurosurgery: a retrospective single-center study. Med Sci Monit. (2021) 27:e932340. doi: 10.12659/MSM.932340, PMID: 34584062 PMC8489250

[12] Yang F Chen H Shan Y Che L Tang Q Hu F. Preventing postoperative moderate- and high-risk pressure injuries with artificial intelligence-powered smart decompression mattress on in middle-aged and elderly patients: a retrospective cohort analysis. Br J Hosp Med. (2024) 85:1–13. doi: 10.12968/hmed.2024.0112, PMID: 39212554

[13] Sumarno AS. Pressure ulcers: the core, care and cure approach. Br J Community Nurs. (2019) 24:S38–s42. doi: 10.12968/bjcn.2019.24.Sup12.S38, PMID: 31804885

[14] Tschannen D Anderson C. The pressure injury predictive model: a framework for hospital-acquired pressure injuries. J Clin Nurs. (2020) 29:1398–421. doi: 10.1111/jocn.15171, PMID: 31889342

[15] Jiang M Cai J Li L Huang X. Comparison of effectiveness of Chinese Association of Operating Room Nurses-Based Nursing Care and Routine Nursing in reducing intraoperative pressure injury in patients undergoing neurosurgery. Adv Skin Wound Care. (2025) 38:210–4. doi: 10.1097/ASW.0000000000000285, PMID: 40131875 PMC12039907

[16] Rivera J Donohoe E Deady-Rooney M Douglas M Samaniego N. Implementing a pressure injury prevention bundle to decrease hospital-acquired pressure injuries in an adult critical care unit: an evidence-based, pilot initiative. Wound Manag Prev. (2020) 66:20–8. doi: 10.25270/wmp.2020.10.2028 33048828

[17] Chaboyer W Latimer S Priyadarshani U Harbeck E Patton D Sim J. The effect of pressure injury prevention care bundles on pressure injuries in hospital patients: a complex intervention systematic review and meta-analysis. Int J Nurs Stud. (2024) 155:104768. doi: 10.1016/j.ijnurstu.2024.104768, PMID: 38642429

[18] Martin S Holloway S. Pressure ulcers: aSSKINg framework study. Br J Community Nurs. (2024) 29:S16–s22. doi: 10.12968/bjcn.2024.29.Sup6.S16, PMID: 38814848

[19] Beeckman D Schoonhoven L Boucqué H Van Maele G Defloor T. Pressure ulcers: e-learning to improve classification by nurses and nursing students. J Clin Nurs. (2008) 17:1697–707. doi: 10.1111/j.1365-2702.2007.02200.x, PMID: 18592624

[20] Zhai C Lin Y. Impact of fast-track rehabilitation nursing on pressure ulcers and postoperative complications in patients with inter-trochanteric fractures: a meta-analysis. Int Wound J. (2024) 21:e14534. doi: 10.1111/iwj.14534, PMID: 38073014 PMC10961043

[21] Yang D Feng R Liu L. Effect of comprehensive nursing based on evidence-based nursing on reducing the incidence of pressure ulcers in patients undergoing posterior orthopedic surgery. Medicine (Baltimore). (2023) 102:e35100. doi: 10.1097/MD.0000000000035100, PMID: 37746975 PMC10519454

[22] Gao MM Wang LP Zhang LL Li YY. The effects of evidence-based nursing interventions on pressure ulcers in patients with stroke: a meta-analysis. Int Wound J. (2023) 20:4069–76. doi: 10.1111/iwj.14298, PMID: 37438328 PMC10681431

[23] Dealey C. Skin care and pressure ulcers. Adv Skin Wound Care. (2009) 22:421–8. doi: 10.1097/01.ASW.0000360255.92357.ad 19713779

[24] Lumbers M. Pressure ulcers: an overview of risk. Br J Nurs. (2017) 26:S49–s50. doi: 10.12968/bjon.2017.26.15.S49, PMID: 28792820

[25] Subrata SA Phuphaibul R. The need for integration nursing theories into pressure ulcer care in the community. Br J Community Nurs. (2022) 27:S6–S10. doi: 10.12968/bjcn.2022.27.Sup12.S6, PMID: 36519485

[26] Dellafiore F Arrigoni C Ghizzardi G Baroni I Conte G Turrini F. Development and validation of the pressure ulcer management self-efficacy scale for nurses. J Clin Nurs. (2019) 28:3177–88. doi: 10.1111/jocn.14875, PMID: 30938908

[27] Asimus M Maclellan L Li PI. Pressure ulcer prevention in Australia: the role of the nurse practitioner in changing practice and saving lives. Int Wound J. (2011) 8:508–13. doi: 10.1111/j.1742-481X.2011.00824.x, PMID: 21827629 PMC7950443

[28] McGraw CA. Nurses' perceptions of the root causes of community-acquired pressure ulcers: application of the model for examining safety and quality concerns in home healthcare. J Clin Nurs. (2019) 28:575–88. doi: 10.1111/jocn.14652, PMID: 30129137

[29] Li J Wu X Li Z Zhou X Cao J Jia Z. Nursing resources and major immobility complications among bedridden patients: a multicenter descriptive study in China. J Nurs Manag. (2019) 27:930–8. doi: 10.1111/jonm.12731, PMID: 30422361

[30] Ham WH Schoonhoven L Schuurmans MJ Veugelers R Leenen LP. Pressure ulcer education improves interrater reliability, identification, and classification skills by emergency nurses and physicians. J Emerg Nurs. (2015) 41:43–51. doi: 10.1016/j.jen.2014.03.005, PMID: 24862184

[31] Athlin E Idvall E Jernfält M Johansson I. Factors of importance to the development of pressure ulcers in the care trajectory: perceptions of hospital and community care nurses. J Clin Nurs. (2010) 19:2252–8. doi: 10.1111/j.1365-2702.2009.02886.x, PMID: 19886875

[32] Buss IC Halfens RJ Abu-Saad HH Kok G. Evidence-based nursing practice: both state of the art in general and specific to pressure sores. J Prof Nurs. (1999) 15:73–83. doi: 10.1016/S8755-7223(99)80078-7, PMID: 10194892

